# Where Do They Come from and Where Do They Go: Candidates for Regulating Extracellular Vesicle Formation in Fungi

**DOI:** 10.3390/ijms14059581

**Published:** 2013-05-02

**Authors:** Débora L. Oliveira, Juliana Rizzo, Luna S. Joffe, Rodrigo M. C. Godinho, Marcio L. Rodrigues

**Affiliations:** 1 The Michael Smith Laboratories, Department of Microbiology and Immunology, University of British Columbia, Vancouver, BC V6T1Z4, Canada; 2 Paulo de Góes Microbiology Institute, Federal University of Rio de Janeiro, Rio de Janeiro 21941-902, Brazil; E-Mails: juju.rizzo@gmail.com (J.R.); lujoffe@gmail.com (L.S.J.); godinho.r@gmail.com (R.M.C.G.); 3 Oswaldo Cruz Foundation–Fiocruz, Centre for Technological Development in Health CDTS), Rio de Janeiro 21040-900, Brazil

**Keywords:** fungal extracellular vesicles, extracellular vesicle biogenesis, exosomes, flippases, GRASP

## Abstract

In the past few years, extracellular vesicles (EVs) from at least eight fungal species were characterized. EV proteome in four fungal species indicated putative biogenesis pathways and suggested interesting similarities with mammalian exosomes. Moreover, as observed for mammalian exosomes, fungal EVs were demonstrated to be immunologically active. Here we review the seminal and most recent findings related to the production of EVs by fungi. Based on the current literature about secretion of fungal molecules and biogenesis of EVs in eukaryotes, we focus our discussion on a list of cellular proteins with the potential to regulate vesicle biogenesis in the fungi.

## 1. Introduction

Fungi are eukaryotic organisms that use complex intracellular trafficking pathways for sorting proteins and lipids to their final cellular sites. Extracellular sites are the final destination of cell wall components, digestive enzymes and, in the pathogenic species, many virulence factors [1–3]. In eukaryotes, the classical secretory pathway has been widely investigated and proteins that undergo this route are synthesized at the endoplasmic reticulum. However, it has been shown that typical intracellular proteins produced by fungi are also found at the extracellular space, playing additional biological functions [4–7]. Efforts to understand how such molecules are driven to the extracellular space led to the finding of a novel secretory mechanism: the release of extracellular vesicles (EVs) by fungal cells. As discussed below, the current literature about fungal EVs still does not allow accurate differentiation between microvesicles, exosomes, ectosomes, apoptotic bodies and other extracellular, vesicle-like structures. Therefore, in this manuscript, we use the term EV to generally define extracellular compartments composed of lipid bilayers forming compartments ranging from 50 to 400 nm in diameter that can be isolated from fungal cultures and/or infected host cells [8–11].

**Figure 1:**
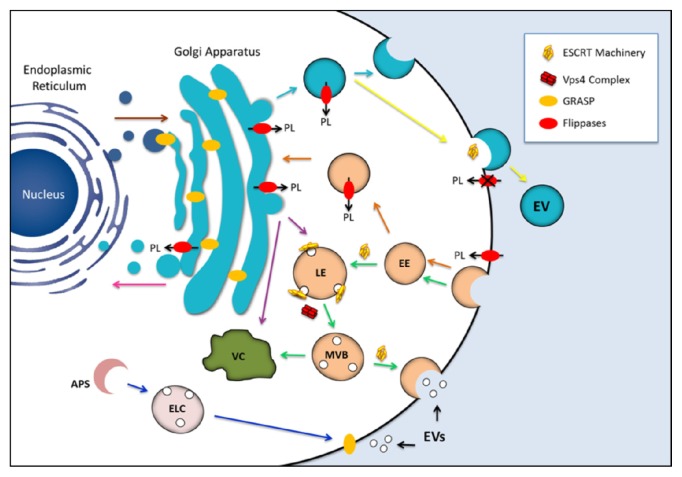
Potential participation of components of the endosomal sorting complex required for transport (ESCRT) machinery, GRASP and flippases in the biogenesis of fungal extracellular vesicles (EVs). The similarities between EVs produced by fungi and mammalian exosomes suggest that ESCRT machinery is required for formation of the fungal compartments (*green arrows*). Maturation of the late endosome (LE) is accompanied by membrane invagination, giving origin to small intraluminal vesicles and multivesicular bodies (MVB). The ESCRT machinery is recycled through the activity of the Vps4 protein complex. MVB may be directed to vacuolar (VC) degradation pathways, but also to fusion with the plasma membrane, releasing exosomes to the extracellular milieu now receiving the name exosomes. GRASP, a regulator of unconventional secretion by mechanisms that are putatively linked to EV release, was first identified as a structural component of the Golgi cisternae. Alternative roles included tethering activity for endosomal or lysosomal compartments and/or regulation of autophagy-related mechanisms (*blue arrows*). GRASP may also localize to the plasma membrane, mediating the release of exosomes to the extracellular space. Finally, GRASP can also participate in docking or fusion events involving vesicles originating at the Golgi, thus facilitating anterograde transport through the early secretory pathway (*brown arrow*). Flippases are involved in vesicle biogenesis through phospholipid translocation across the lipid bilayers. These enzymes can regulate endocytosis at the plasma membrane level (*orange arrows*) and also drive the formation of exocytic vesicles (*light blue arrows*). Flippases can also participate in protein trafficking between the *trans*-Golgi network and endosomal compartment or between the *trans*-Golgi network and vacuoles (*purple arrows*). It has been also proposed that flippases may regulate the retrograde transport pathway from the Golgi apparatus to the ER (*pink arrow*), as well as vesicle budding at the plasma membrane level (*yellow arrow*). The possibility that cellular pathways regulated by endosomal proteins, GRASPs and flippases are interconnected cannot be ruled out, as previously described for other unconventional secretory pathways [165]. Most of the mechanisms proposed here have been implicated with the physiology of yeast cells, although they also participate in pathways required for molecular degradation and / or export in other eukaryotes. (PL) phospholipid; (EE) early endosome; (LE) late endosome; (MVB) multivesicular bodies; (APS) autophagosome; (ELC) endosomal/lysosomal compartment; (VC) vacuole.

Fungal EVs have been analyzed by a number of approaches that revealed important morphological features and complex composition [6,9–13]. Models of stimulation of host cells *in vitro* and *in vivo* with fungal EVs clearly demonstrated that these compartments are immunologically active and have the potential to regulate key pathogenic steps during fungal infections [6,10,11,14]. On the other hand, mechanisms required for biogenesis of fungal EVs still consist of an open field with more questions than answers [11,15,16]. In the next sections, we summarize the current knowledge on the structural and functional properties of fungal EVs. We also discuss pathways with the potential to regulate EV biogenesis in fungi, with focus on three classes of proteins with still poorly explored physiological roles in pathogenic fungi: endosomal proteins, flippases and Golgi reassembly and stacking protein (Figure 1).

## 2. An Overview on Molecular Traffic and EV Release

EV export has been proven to be essential to a variety of cellular systems. For instance, in the Prokaryotes, the ability of Gram-negative bacteria to produce outer membrane-derived vesicles (OMVs) carrying virulence factors has been documented in detail [17–19]. Gram-positive bacteria including *Staphylococcus aureus* and *Bacillus anthracis* were also demonstrated to produce EVs related to virulence, although their cellular origin is so far poorly understood [20,21]. Comparative genomic analysis revealed that members of the Archaea domain produce proteins responsible for membrane remodeling and vesicle formation [22]. In fact, EVs were isolated from members of the Sulfolobales and Thermococci orders as part of the response to viral infections [23,24].

In mammalians, the release of vesicles to the extracellular space has been widely studied for the past two decades. Mammalian cells produce different types of EVs, including secretory lysosomes, multi-vesicular bodies (MVB)- derived exosomes or microvesicles shed from the plasma membrane [25–27]. Exosomes are probably the most well studied type of mammalian EVs. These compartments were originally reported to be involved in reticulocyte maturation [28], but it is now clear that they are involved in multiple biological events. Exosomes are required for antigen presentation [29,30], neuronal communication [31] viral spreading [32,33], defense against microbial pathogens and cancer cells, [34,35] and tumor metastasis [36]. Exosome-like EVs were also described as pathogenesis-related transport vehicles in helminthes [37] and protozoans [38,39]. Finally, plant cells have also been described as producers of exosome-like vesicles [40]. Together, these studies indicate that the release of membranous vesicles to the extracellular space is a biological process conserved among a variety of cell types and a common property of cellular systems in nature.

## 3. Fungal EVs

In the Fungi, production of EVs was initially suggested in early reports [41,42], but fully characterized in the pathogenic yeast *Cryptococcus neoformans* only five years ago [8]. From the seminal discovery in the *C. neoformans* until now, fungal EVs were identified in at least eight additional fungal species [9–11,14]. The hypothesis that EVs by are produced by additional fungal species is supported by different reports describing vesicle-like structures as components of regular physiologic events [43] or products of viral infections [44].

The most remarkable feature of *C. neoformans* is the presence of a polysaccharide capsule coating the cell surface. The capsule is mostly composed of glucuronoxylomannan (GXM), a high molecular weight fibrillar polysaccharide that corresponds to the main virulence factor of *C. neoformans* [45–47]. The mechanisms by which GXM contribute to *C. neoformans* virulence potentially involve its effective immunossupressive and anti-phagocytic properties [48].

Differently from other cell wall polysaccharides produced by microbes, GXM synthesis occurs at the intracellular level, in processes that are apparently Golgi-dependent [49]. The Golgi apparatus is the organelle responsible for the sorting of proteins to different cellular compartments, depending on specific glycosylation signatures [50]. Indeed, all molecules that engage the classical biosynthetic secretory pathway ought to pass through the Golgi apparatus in order to reach the extracellular space [50]. In *C. neoformans*, GXM was distributed into Golgi-derived intracellular vesicles [49]. Independent studies have shown, through the use of different electron microscopy techniques, that vesicle-like, GXM-containing compartments are present in the periplasm and through the cell wall [8,13,41,42,51]. GXM-containing vesicles were also isolated from culture supernatants, using protocols that were adapted from studies with mammalian exosomes [8]. Similar approaches allowed the identification of fungal EVs in *Histoplasma capsulatum*, *Saccharomyces cerevisiae*, *Sporotrix shenkii*, *Candida albicans*, *Candida glabrata*, *Paracoccidioides brasiliensis*, and *Malasezzia sympodialis* [9–11,14].

Compositional studies revealed that fungal EVs carry a complex mixture of different macromolecules. Proteomic characterization of vesicle fractions in *C. neoformans*, *H. capsulatum*, *S. cerevisiae* and *P. brasiliensis* [6,9,11,52] revealed proteins common to all four species. Strikingly, the EV proteome revealed cytoplasmic, plasma membrane, mitochondrial, vacuolar and even nuclear proteins [6,9,11,52]. This great variety in protein composition and functions supported a vast literature related to the presence in the Fungi of proteins localized to different cellular compartments [2,53]. Biologically active enzymes [6,54], sterols [8,11,16], phospholipids [9,13,16], polysaccharides [8,10] and pigments [6,54] were also characterized as components of fungal EVs. This great molecular diversity suggested that fungal EVs would likely interfere with the host immune response, as previously described for mammalian exosomes and EVs produced by parasites [34,38,39,55]. In fact, *P. brasiliensis* vesicles were demonstrated to contain highly immunogenic-galactosyl epitopes [10]. Vesicular proteins produced by *H. capsulatum* and *C. neoformans* were recognized by antibodies in sera from infected patients, but not from healthy individuals [6,9].

The results described above in combination with the description of virulence-related molecules in fungal EVs [6,8–10,52] were suggestive that these compartments might interfere with the physiology of host cells. Indeed, *in vitro* studies using macrophage-like cells and peripheral blood mononuclear cells (PBMCs), as well as the evaluation of the role of fungal EVs during mice cryptococcosis, supported this hypothesis [11,14]. *C. neoformans* and *M. sympodialis* EVs activated effector immune cells, ultimately inducing cytokine production [11,14]. The activation of macrophages by *C. neoformans* vesicles was accompanied by an augment in the phagocytosis index of yeast cells, as well as by a more effective fungicidal activity [11]. *C. neoformans* EVs also promoted enhanced passage across the blood-brain barrier by the fungus *in vitro* and increased the efficacy of cryptococci to colonize the brain of infected mice [56]. Together, these results suggest that EVs might actively participate in fungal pathogenesis.

The studies summarized above clearly demonstrate the advances during the last five years in the understanding of compositional and immunobiological aspects of fungal EVs. An analogous analysis of the progress made on the understanding of how fungal EVs are formed, however, suggests a completely different scenario. Studies on vesicle biogenesis, so far, addressed the involvement of both conventional and unconventional secretory pathways in formation of fungal EVs, but they were all unsuccessful in connecting the typical regulators of secretion with EV production by fungi [11,57,58]. In studies with *C. neoformans* and *S. cerevisiae*, the conventional secretory regulators Sec4, Sec1, Sec6 and Sec32, as well as the vacuolar protein-sorting (Vps) proteins Vps23 and Snf7, were all demonstrated to partially affect EV composition and /or their kinetics of extracellular release, but none of these proteins were consistently associated with blocking of EV formation [11,57]. In addition, density gradient fractionations demonstrated the existence of vesicle sub-populations differing in their molecular content, suggesting that more than one pathway might be implicated in vesicle generation [13]. Therefore, it is now clear that additional regulators of vesicle formation might be investigated to improve the understanding of the mechanisms involved in EV biogenesis in fungi. Based on the current literature of EV formation in mammalian cells, we will discuss here different and still unexplored cellular pathways with the potential to participate in the biogenesis of fungal EV.

## 4. Candidates for Regulating the Formation of Fungal EVs

### 4.1. Exosome Regulators

The term exosome was first used by Trams and colleagues to describe 50 nm diameter vesicles observed in several cultures of normal and neoplasic cells [59]. Harding and colleagues further used electron microscopy techniques to demonstrate that small, luminal vesicles of endosomal compartments could be released to the extracellular space upon fusion between endosomal and plasma membranes [60,61].

Exosomes can be identified by morphological and biochemical criteria. Their dimensions usually correspond to those observed for the vesicles present in the endosomal compartments from where they originate (40–100 nm diameter). Exosomes are usually isolated from culture supernatants using ultracentrifugation procedures [62]. This method, however, frequently fails to distinguish exosomes from other small vesicles and macromolecular aggregates, implying that additional approaches are required for their characterization. These approaches may include immunoblotting analysis, mass spectrometry, and different imaging techniques [63]. Size distribution analysis by dynamic light scattering and high throughput flow cytometric approaches [64–66] have also been successfully applied for exosome characterization. Exosomal membrane composition reflects mechanisms of biogenesis. Proteins of exosomal membranes contain endosomal molecules, including Rab GTPases, SNAREs, anexin and flotilin, in addition to plasma membrane proteins, including tetraspanins [67] and lipid raft-related proteins [68,69]. Lipidomic studies of exosomal membranes indicated cholesterol, sphingomyelin, and hexosylceramides as key hydrophobic components [69–72].

As mentioned before, exosomes are originated from the membrane of endosomal compartments. The invagination of the late endosome membrane forms small vesicles within its lumen giving origin to what was first described by Porter and Palade as “large vesicles with smaller vesicles inside”[73]. A few years later, Sotelo and Porter [74] proposed classification of these structures as multivesicular bodies (MVB). It is now well known that MVB formation relies on the functions of the endosomal sorting complex required for transport (ESCRT), which refers to a series of cytosolic protein complexes called ESCRT-0, I, II and III. The ESCRT machinery was first described in the model yeast *S. cerevisiae* [75–77]. In this organism the products of the vacuolar protein sorting (*vps*) class E genes assemble into five different units, the above-mentioned ESCRT-0, I, II and III and Vps4 [75–80]. Each one of these protein complexes plays specific tasks not only in MVB formation but also in cell shrinking and viral budding. These latter aspects were reviewed elsewhere [81,82]. We will discuss below the molecular aspects of MVB formation and its possible relationship with the formation of EVs in fungi.

Although the ESCRT machinery has been studied in detail in the last two decades, many of the aspects by which it regulates formation of intraluminal vesicles (ILVs) are still obscure. It is generally accepted that the process begins with the recognition of ubiquitinated cargo proteins by ESCRT-0. This protein complex is composed by two subunits, Vps27 and Hse1, both capable of cargo recognition via ubiquitin interaction motif (UIM) and VHS domains (acronym derived from occurrence in the proteins Vps27, Hrs and STAM). The presence of a zinc finger motif on Vps27 ensures the affinity of the complex by lipid membranes through interaction with phosphatidylinositol 3-phosphate (PtdIns3P) [78,83].

The next step toward MVB formation is the recruitment of the second protein complex, ESCRT-I. This complex, which is composed by the subunits Vps23, Vps28, Vps37 and Mvb12, interacts with the ESCRT-0 PTAP-like motif (formed by aminoacids *p*roline, *t*hreonine, and *a*lanine), contained on Vps27 via its Vps23 subunit [75,84,85]. ESCRT-I is also capable of cargo recognition as it contains an ubiquitin E2 variant (UEV) domain on Vps23 and also an ubiquitin-binding domain (UBD) on MVB12 [75,84,86,87].

ESCRT-II is composed of Vps22, Vps25 and Vps36 [76,88]. The last protein is responsible for the interaction between this complex via its GRAM-like ubiquitin-binding in Eap45 (GLUE) domain on ESCRT-1 Vps28 C-terminus [89,90]. ESCRT-II is “Y” shaped and formed by copies of Vps25, which interact with high affinity with Vps20, forming a multiple arm-like structure [91,92].

Vps20 is one of the constituents of ESCRT-III complex, along with Snf7, Vps24 and Vps2 [77]. This is the only ESCRT component that does not form stable cytoplasmic complexes. Upon interaction of Vps20 with Vps25, the former begins recruiting monomers of Snf7, which interact with each other by forming long filament-like polymers that are thought to be capped by molecules of Vps24. When elongation reaches the endosome, Vps24 recruits Vps2, completing formation of ESCRT-III [92–94]. The accessory molecules Bro1/Alix (BCK1-like resistance to *o*smotic shock protein-1/apoptosis-linked gene-2 interacting protein *X*) and Doa4 (degradation *o*f alpha-4), which are both recruited by Snf7, regulate cargo deubiquitination [95,96].

Vps4, a component of the ESCRT-III machinery, is a class I AAA ATPase [79,97]. The dissociation of ESCRT-III from the membrane of the MVB requires energy that is provided by the Vps4-Vta1 complex [98]. This complex associates with the MIM (missing in metastasis) domains in ESCRT-III via *N*-terminal MIT (microtubule interacting and trafficking) domains [79,99]. Another possible function for the Vps4-Vta1 complex is the modulation of ESCRT-III activity through its multiple interactions via MIT domains with several Snf7 molecules and accessory proteins, like the Did2/Ist1 complex, possibly acting as an endosomal anchor for the whole Vps4 machinery [100,101].

Although the steps of ESCRT recruitment are well known, the mechanisms by which invagination of the endosomal membrane results in vesicle luminal release are still obscure. Two models explaining this phenomenon have been proposed. In the first model, nominated Snf7 oligomerization, each Vps25 arm of ESCRT-II can recruit one Vps20, which recruits two Snf7 molecules. The filament resulting from the elongation of the Snf7 oligomer associated with Vps24 and Vps2 would induce membrane deformation, causing ILV release [102]. Accordingly, systems at which mutations generating a “one armed” ESCRT-II were induced showed Snf7 elongation but failure to generate ILVs. On the other hand, Snf7 overexpression led to the formation of ILVs with larger, abnormal diameter (up to 360 nm) [103]. Apparently, the control of Snf7 oligomerization relies on the activity of Vps24 and Vps2, which stops Snf7 addition and recruits the Vps4-Vta1 complex for ESCRT-III release prior to ILV formation [81,103]. The second model proposed to explain the release of ILVs has been nominated the Snf7 dome. According to this model, Vps24-Vps2 spirals of decreasing diameter would distribute inside the forming ILV neck limited by Snf7 oligomers, forming a protein cap that prevents the loss of ILV cargo. The created membrane curvature would then facilitate membrane scission and vesicle release [104,105].

Although the involvement of the ESCRT machinery with MVB/vesicle formation is clear, the existence of other mechanisms with similar functions cannot be discarded. For example, in mammalian cells, ILV formation is unaffected even in the absence of central ESCRT components [106,107], suggesting alternative, ESCRT-independent mechanisms of MVB formation. In fact, *in vitro* studies demonstrated that lysobisphosphatidic acid (LBPA) drives vesicle budding inside LBPA-containing liposomes in processes that involve pH gradients across the membrane [108]. In addition, ceramide formation has been also linked to membrane deformation and budding of ILVs [107].

MVB-like structures fusing with the plasma membrane have been observed in *C. neoformans* [41,109]. In addition, fungal EVs manifested morphological characteristics and protein composition with similarity to that of mammalian exosomes. Deletion of *SNF7* in *S. cerevisiae* resulted in altered composition of EVs [11], suggesting that the MVB-related machinery might me related to the biogenesis of exosome-like structures in fungi. Therefore, most of the above-mentioned proteins required for MVB formation could represent important regulators of formation of fungal EV.

### 4.2. Flippases

Lipid asymmetry is critical to many cellular functions, including regulation of membrane curvature and budding of vesicles [110–112]. The correct membrane asymmetry relies on the activity of transmembrane proteins belonging to type 4 subfamily of P-type ATPases, the flippases. P-type ATPases represent an ubiquitous family of proteins that facilitate the ATP-dependent transport of ions and heavy metals across biological membranes [113]. Based on phylogenetic analysis, this large family is divided into five major subfamilies, each of them with unique affinities for their specific substrates [114]. Within the P-type ATPase family, P4-ATPases are responsible for phospholipid transport. Based on this biological activity, they were nominated aminophospholipid translocases (APTs or APLTs) [113–118].

The primary biological activity of APTs is to promote lipid translocation. APTs preferentially transfer phosphatidylserine (PS) and phosphatidylethanolamine (PE) from the external face (extracytoplasmic) of the plasma membrane to its inner (cytoplasmic) side [115,119–121]. An important and conserved feature of P4-ATPases is their ability to form heteromeric complexes with members of the Cdc50 protein family [122–125]. The exact role played by Cdc50p in association to P4-ATPases is not yet fully understood. It has been proposed, however, that molecules belonging to the Cdc50 protein family participate in the substrate specificity of flippases [126].

Multiple members of the P4-ATPase family are distributed into eukaryotic genomes [127]. Prokaryotes, however, lack P4-ATPases. In *S. cerevisiae*, five aminophospholipid translocases (Drs2p, Dnf1p, Dnf2p, Dnf3p and Neo1p) have been described and all of them are directly involved with vesicular traffic of macromolecules. Drs2p and Dnf3p localize to the late Golgi compartment and potentially to endosomal membranes, whereas Dnf1p and Dnf2p localize primarily to the plasma membrane [128–130]. Neo1p is apparently distributed into cellular sites that include both endosomes and the Golgi apparatus, which might explain its participation in the retrograde transport pathway [131,132].

A direct association between yeast flippases and vesicular mechanisms of traffic has been established after the observation that mutants with defects in the activity of Dnf1p, Dnf2p and Drs2p lack functionality in both endocytic and exocytic pathways [130,133–136]. In *Caenorhabditis elegans*, the putative flippases TAT- 1and TAT-5 were demonstrated to regulate endocytic pathways [137] and formation of extracellular vesicles [138], respectively. Studies in other organisms including *Arabidopsis thaliana*, *Leishmania donovani*, and also mammals indicated that flippases have essential activities in the early mechanisms of vesicle biogenesis, vesicular traffic and/or maintenance of membrane integrity [111,123,125]. In erythrocytes, stimulation of the activity of APTs induced formation of endocytic vesicles [139,140]. A generally accepted hypothesis supporting the findings described above is that flippases might affect vesicle formation as a consequence of enrichment of specific phospholipids in one side of bilayered membranes [112], leading to membrane curvature. This mechanism has been nominated as the “bilayer couple mechanism” of membrane reshaping [141,142].

Small vesicles and tubular structures mediate protein transport between organelles of the secretory and endocytic pathways [50]. Several proteins control mechanisms of biogenesis and traffic of these vesicles, including those belonging to the coat protein complex (COP), adaptor proteins (APs), ADP-ribosylation factor (ARF), and its guanine-nucleotide-exchange factors (GEFs), among others. Basically, COPII-coated vesicles bud from the endoplasmic reticulum (ER) to deliver cargo to the Golgi, and COPI-coated vesicles mediate retrograde transport from the Golgi back to the ER. Clathrin associates with organelle-specific adaptor proteins (APs) to form vesicles from the *trans*-Golgi network (TGN), endosomes and the plasma membrane, mediating multiple transport pathways between these compartments [50]. The small, GTP binding protein ADP-ribosylation factor (ARF), and its guanine-nucleotide-exchange factors (GEFs) and GTPase activating proteins, regulate the assembly of COPI, clathrin-adaptor and AP-3 coat proteins on the Golgi and/or endosomes [143].

Genetic studies in yeast have implicated Drs2p, a flippase found in the late *trans-*Golgi network (TGN) of *S. cerevisiae*, in ARF and clathrin-dependent formation of transport vesicles [128,144]. The connection between Drs2p with Arfp suggests that this protein is important in processes of vesicular budding at the TGN, indicating that P4-ATPases might be part of the normal protein trafficking machinery. Indeed, studies with *S. cerevisiae* revealed that proteins whose traffic is related to Arf and/or clathrin vesicles in the TGN had their secretory efficacy altered after flippase deletion. Chitin synthase III (Chs3p) is a cargo protein in yeast that requires AP-1/clathrin for trafficking in the TGN–endosomal system. Loss of Drs2p affects Chs3p trafficking in a process that culminates with altered cell surface expression and increased trafficking of the protein into the late endosomes [136,145]. *Drs2* mutants also exhibited a defect in generating exocytic vesicles carrying invertase and acid phosphatase [144]. These vesicles require clathrin for their formation and at least a portion of them are clathrin-coated [146,147]. A *drs2 Δdnf1Δ* double mutant exhibited an even lower efficacy in transport pathways required for the export of alkaline phosphatase protein and carboxypeptidase Y [129]. This observation suggests that Drs2p and Dnf1p are functionally redundant in their ability to regulate pathways of vacuolar protein transport. Moreover, a *dnf1Δ dnf2Δ dnf3Δ* triple deletion mutant exhibited defects in the endosome to TGN recycling pathway [129]. ARF, clathrin, and/or adaptins (AP-1, AP-3 and GGAs) have been implicated in each of the pathways requiring Drs2/Dnf proteins for normal functioning. Finally, Neo1p is required for protein transport in early mechanisms of the conventional secretory pathway, as well as in Golgi-dependent glycosylation [131]. For instance, Rer1, a protein that cycles between the ER and Golgi complex in COPI and COPII vesicles, is mislocalized to the vacuole in *neo1* temperature sensitive mutants [131]. Altogether, these results suggest that flippases are deeply involved in different secretion mechanisms requiring vesicles.

Flippases have been reported to regulate important physiological and pathogenic events in different eukaryotic models. In *Magnaporthe grisea*, a rice fungal pathogen, two putative APTs, *MgAPT2* and *MgPDE1*, were implicated in the development of invasive hyphae and also in vesicular export of pathogenesis-related proteins [148,149]. In the fungal pathogen *C. neoformans*, mutants lacking expression of the APT1 flippase manifested attenuated virulence and defects in responding to oxidative and nitrosative stresses. The *apt1* mutants had increased sensitivity to brefeldin A and monensin, which interfere with the ER-Golgi trafficking [150]. These findings are probably related to the fact that *C. neoformans* uses a vesicular network of traffic to export polysaccharides [8,49], which is determinant for pathogenicity. In plant studies using the *Arabidopsis thaliana* model, deletion of the putative flippase ALA3 affected polysaccharide secretion and formation of TGN-derived vesicles [123]. These results strongly suggest that flippases might be an important part of the regulation of vesicular export mechanisms of proteins and polysaccharides in eukaryotic cells.

### 4.3. Golgi Reassembly Stacking Protein (GRASP)

The Golgi apparatus is typically formed by one or more layers of associated membranous discs called cisternae [151]. As previously discussed by Ramirez and colleagues, the individual layers of the Golgi are dispersed in the cytoplasm of fungi, plants and lower eukaryotes [152]. In most vertebrates, these layers are laterally connected to form a complex and bulky structure.

To maintain the flow of proteins that are continuously transported inside the Golgi apparatus, great membrane flexibility is required, but the existence of mechanisms that maintain the basic structure of the organelle are also essential. Early studies demonstrated protein connections between the cisternae, a seminal observation that has been confirmed in more recent studies [27,152]. GRASP (Golgi reassembly and stacking protein) is supposed to be one of these proteins [153]. Two isoforms of GRASP (GRASP55 and GRASP65) are present in vertebrates and a single gene is found in other eukaryotes, except plants [154]. GRASPs were also suggested to participate in the tethering of vesicles destined to fuse with the Golgi apparatus [153,155]. Other studies, however, suggested that GRASPs might have additional, non-structural roles that are independent of their primary functions. For instance, depletion of GRASP in *Drosophila*, *S. cerevisiae* and mammalian cells had no significant effects on cisternal stacking or general protein secretion [153,156–160]. Plant cells, which lack GRASP-related genes, have perfectly stacked Golgi cisternae. On the other hand, GRASP has been described as a key regulator of unconventional mechanisms of protein secretion [161]. These observations suggest that, although the most essential functions of GRASP in eukaryotes are still obscure, this class of proteins might be essential for non-classic mechanisms of protein secretion.

In *Dictyostelium discoideum*, deletion of the single gene encoding GRASP resulted in defective secretion of acyl-coenzyme A binding protein (AcbA), which lacks the signal peptide required for engagement in the conventional secretory pathway [161]. AcbA-containing vesicles accumulated beneath the plasma membrane, suggesting a role for GRASP in unconventional, vesicular mechanisms of secretion of this cytoplasmic protein [162]. Similar findings were described in *S. cerevisiae* and *Pichia pastoris* [163,164]. In the former, GRASP has been found to interact with the COPII coat proteins Sec23 and Sec24, suggesting a role in the docking or fusion of COPII-coated vesicles at the Golgi, thus facilitating anterograde transport through the early secretory pathway [156].

The alternative roles of GRASP proposed in different studies suggest complex functions that would require additional cellular sites for the protein, besides the Golgi apparatus. It has been proposed that GRASP might mediate direct transport of AcbA across the plasma membrane by still unknown mechanisms that might require a plasma membrane distribution [154]. As a plasma membrane protein, GRASP could also act as a tether for endosomal or lysosomal compartments that have engulfed cytoplasmic AcbA in exosomes, or to which AcbA had been delivered by an autophagy-like process [165]. This cellular distribution and related functions would be in agreement with the observation that, during *D. melanogaster* development, GRASP is required for the delivery of α-integrin to the plasma membrane through a Golgi-independent manner [166]. Although most of these hypotheses still require experimental proof, a realistic perspective is that GRASP might be required for the vesicular traffic of a number of molecules in eukaryotic cells.

The functions of GRASP in eukaryotes apparently include the traffic of polysaccharides to the extracellular space, as demonstrated in the *C. neoformans* model [58]. Polysaccharide secretion in this fungus was initially linked to proteins with homology to Sec4 and Sec6 [49,57], two key regulators of conventional mechanisms of exocytosis in other models [167]. Although polysaccharide secretion was affected in the mutants lacking expression of the *SEC4* and *SEC6* orthologs, capsule assembly appeared normal in those cells [49,57]. This observation led to the hypothesis that polysaccharide secretion might also require regulators of unconventional secretion pathways. The fact that extracellular vesicles produced by *C. neoformans* are loaded with capsular polysaccharides [8,13] and the possibility that GRASP is required for mechanisms of secretion involving EVs in yeast cells [154,163,164] support this hypothesis. In this regard, a general analysis of putative defects in protein secretion in the *graspΔ* mutant of *C. neoformans* and its potential correlation with polysaccharide export might be a promising approach to correlate unconventional mechanisms of secretions of glycans and proteins in this fungus.

Deletion of the single GRASP ortholog of *C. neoformans* did not affect the traffic of pigments and urease [58], which are surface and extracellular molecules regulating key pathogenic steps in this fungus [168,169]. Deletion of the GRASP ortholog, however, profoundly affected polysaccharide secretion and, consequently, capsule formation [58]. The defective polysaccharide secretion in the mutant lacking GRASP resulted in a hypovirulent phenotype, associated with an increased susceptibility to the antimicrobial activity of macrophages [58,168]. It remains unknown why lack of GRASP in *C. neoformans* results in decreased polysaccharide secretion. GRASP could regulate the export of polysaccharide-containing extracellular vesicles to the milieu by the mechanisms proposed for unconventional secretory steps in *S. cerevisiae*, *D. discoideum*, *D. melanogaster* and *P. pastoris* [155,160,162–165]. An alternative possibility would derive from the fact that the glycosyltransferases required for polysaccharide and glycoprotein synthesis in eukaryotes are essentially Golgi-associated. Since the *C. neoformans* mutant lacking the GRASP gene showed abnormal Golgi morphology [58], dysfunctions in intracellular glycan synthesis might be expected. *C. neoformans* GRASP could be also required for loading GXM into secretory vesicles, although this hypothesis still requires experimental proof. Finally, considering that the GRASP orthologs of *S. cerevisiae* and *P. pastoris* are supposed to be functional at the tER-Golgi interface [170], we speculate that lack of GRASP could also affect the functionality of cellular sites playing central functions within the secretory machinery.

## 5. Concluding Remarks

Studies based on the characterization of fungal EVs produced by mutants with defects in conventional or unconventional secretory pathways have failed in demonstrating the mechanisms required for biogenesis of these extracellular membrane compartments [11,167]. This observation makes clear the need of identification of additional targets with the potential to regulate EV biogenesis in the Fungi. Based on the current literature, we propose that proteins related to mechanisms of EV biogenesis in other eukaryotes (ESCRT machinery, flippases and GRASP) whose functions in EV formation in fungi are still unexplored could be excellent targets for future studies aiming at understanding some of the mechanisms required for regulation of this interesting phenomenon, as summarized in Figure 1.
